# Glucose-Modulated Mitochondria Adaptation in Tumor Cells: A Focus on ATP Synthase and Inhibitor Factor 1

**DOI:** 10.3390/ijms13021933

**Published:** 2012-02-10

**Authors:** Rossana Domenis, Elena Bisetto, Davide Rossi, Marina Comelli, Irene Mavelli

**Affiliations:** 1 Department of Medical and Biological Sciences, University of Udine, p.le Kolbe 4, 33100 Udine, Italy; E-Mails: rossana.domenis@uniud.it (R.D.); elena.bisetto@uniud.it (E.B.); davide.rossi@uniud.it (D.R.); marina.comelli@uniud.it (M.C.); 2 M.A.T.I. Centre of Excellence, University of Udine, p.le Kolbe 4, 33100 Udine, Italy

**Keywords:** ATP synthase, inhibitory factor 1, metabolic adaptation, tumor bioenergetics, hyperglycemia, aglycemia, mitochondria, oxidative phosphorylation, HepG2

## Abstract

Warburg’s hypothesis has been challenged by a number of studies showing that oxidative phosphorylation is repressed in some tumors, rather than being inactive *per se*. Thus, treatments able to shift energy metabolism by activating mitochondrial pathways have been suggested as an intriguing basis for the optimization of antitumor strategies. In this study, HepG2 hepatocarcinoma cells were cultivated with different metabolic substrates under conditions mimicking “positive” (activation/biogenesis) or “negative” (silencing) mitochondrial adaptation. In addition to the expected up-regulation of mitochondrial biogenesis, glucose deprivation caused an increase in phosphorylating respiration and a rise in the expression levels of the ATP synthase β subunit and Inhibitor Factor 1 (IF1). Hyperglycemia, on the other hand, led to a markedly decreased level of the transcriptional coactivator PGC-α suggesting down-regulation of mitochondrial biogenesis, although no change in mitochondrial mass and no impairment of phosphorylating respiration were observed. Moreover, a reduction in mitochondrial networking and in ATP synthase dimer stability was produced. No effect on β-ATP synthase expression was elicited. Notably, hyperglycemia caused an increase in IF1 expression levels, but it did not alter the amount of IF1 associated with ATP synthase. These results point to a new role of IF1 in relation to high glucose utilization by tumor cells, in addition to its well known effect upon mitochondrial ATP synthase regulation.

## 1. Introduction

The most popular paradigm of metabolic remodelling describing the occurrence in tumor cells (the Warburg effect) comprises an increase in glucose uptake, an enhancement of glycolytic capacity, high lactate production, and the absence of respiration (despite the presence of high oxygen concentrations) caused by irreversible damage to mitochondrial function [1]. Over the last decade, this “metabolic dogma” has been re-evaluated in light of various bioenergetic studies that have emphasized the variability existing between and even within cancer types as regard to the mechanisms and substrates preferentially used for deriving vital energy [2]. Nowadays, Warburg’s “aerobic-glycolysis” hypothesis has been challenged by a growing number of studies, showing that mitochondria, in some tumor cells, are not inactive *per se*, but operates at low capacity, repressed by the presence of glucose, demonstrating the importance of a dynamic view of tumor bioenergetics [3].

Several mechanisms that directly promote the inhibition of mitochondrial function have been proposed, even if the molecular basis of this phenomenon is still unknown [4]. Moreover, it has been demonstrated that some tumor cell lines can switch from aerobic glycolysis to oxidative phosphorylation (OXPHOS) under glucose-limiting conditions [5–8] via alterations in the morphology of the mitochondrial network and the up-regulation of mitochondrial mass and respiratory chain protein expression levels. It has also been demonstrated that cancer cells behave differently to normal cells when adapting their bioenergetics to microenvironmental conditions (hypoxia and aglycemia) [6,9]; thus, this difference in the tolerance of cancer cells to their environments could be important for optimizing anti-cancer treatment strategies. Indeed, a recent review discussed how the pharmaceutical intervention of cellular energy metabolism could render tumor cells more susceptible to anti-cancer treatments [4]; different possibilities were indicated depending on the predominant ATP-generating pathways concerned. In addition to the well known suppression of the glycolytic pathway, it has been proposed that shifting a cell’s metabolism to glucose oxidation may reverse the glycolytic phenotype, thereby restoring the cell’s sensitivity to apoptotic inducers and opening up another effective therapeutic strategy. Recently, it was reported that the overexpression of the mitochondrial frataxin protein [10] or attenuation of lactate dehydrogenase A [11] increases mitochondrial energy metabolism in tumor cell lines, resulting in the suppression of tumor growth. In conclusion, understanding the molecular mechanisms involved in the bioenergetic mitochondrial adaptation/silencing of tumor cells and the limits of this phenomenon is of fundamental importance for defining this form of plasticity and for understanding tumor tolerance to changes in nutrient composition. This is especially the case when considering that different mitochondrial backgrounds may confer a survival advantage to tumors cells in response to chemotherapy [12].

In this study, the human hepatocellular carcinoma cell line HepG2 was used; the mitochondrial bioenergetic profile of which has already been characterized with regard to its differentiation/growth tumor grade [13]. Cells were cultivated in the presence of different concentrations of glucose with the aim of inducing “positive” mitochondrial plasticity in aglycemia conditions, as well as “negative” adaptation to hyperglycemia. This strategy was based on the knowledge that high glucose concentrations are involved in the silencing of mitochondria in tumors, whereas glucose deprivation is involved in their activation/biogenesis. The study focuses on the effects of these conditions upon (i) ATP synthase, as the impact of metabolic adaptation on the supramolecular organization of ATP synthase has not yet been described; and (ii) Inhibitor Factor 1 (IF1), since it has now been recognized that IF1 function is not limited to its role as the physiological inhibitor of ATP synthase, but that it also constitutes a key component in the metabolic switch of tumor cells [14–16].

## 2. Results and Discussion

### 2.1. Cell Proliferation and Mitochondrial Content/Biogenesis

Cell proliferation rates (*i.e.*, doubling times) of HepG2, grown in the three different culture conditions, were compared. HepG2 cells divided faster in the high glucose (25 mM) medium (doubling time: 19 ± 0.8 h) than in the intermediate (11 mM) glucose condition (doubling time: 30.7 ± 0.5 h). In galactose medium, the doubling time was approximately two times longer (50 ± 1.2 h). No significant change in cell morphology or cell viability was observed in HepG2 grown under the three different conditions.

**Figure 1:**
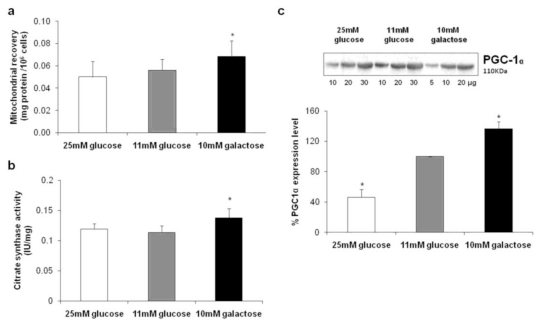
Analysis of mitochondrial content and biogenesis (**a**) Mitochondrial recovery was expressed as mg of mitochondrial protein per 10^6^ cells. Values are means of seven different experiments (bars represent s.d.), * *p* < 0.005 compared with HepG2 cultivated in the presence of 11 mM glucose; (**b**) The enzymatic measurement of citrate synthase (CS) activity was performed spectrophotometrically on total cell homogenates and expressed as IU/mg of total protein. Values are means of three different experiments (bars represent s.d.), * *p* < 0.001 compared with HepG2 cultivated in the presence of 11 mM glucose; (**c**) The content of peroxisome proliferator-activated receptor-γ coactivator-1α (PGC-1α) was determined by Western blot analysis on total cell homogenates. Different quantities of protein were separated by SDS-PAGE, transblotted, and identified with specific antibodies. For quantification purposes and as loading control, a linear relationship was verified between the band intensities (densitometric analysis) and the protein quantities loaded into the gel to ensure non-saturating conditions and high reproducibility. Values were inferred on the basis of the slope of the straight line and are means of three different experiments (bars represent s.d.), * *p* < 0.05 compared with HepG2 cultivated in the presence of 11 mM glucose taken as 100%.

Cell mitochondrial contents were evaluated by estimating the protein recovery in the crude mitochondrial fractions and are reported in Figure 1a. We documented a higher mitochondrial content in HepG2 cultivated in 10 mM galactose with respect to that observed when the cells were cultivated in medium with 11 mM or 25 mM glucose, both of which were similar. This difference was further validated in evaluating the mitochondrial mass by assaying CS activity in total cell homogenates. The results, reported in Figure 1b, confirm a significant increase in mitochondrial content of HepG2 grown under aglycemic conditions.

We also determined the expression level of a key regulator of mitochondrial biogenesis, the peroxisome proliferator-activated receptor-γ coactivator-1α (PGC-1α), by Western blot analysis on cell lysates. The results, shown in Figure 1c, indicate an increase in the expression level of PGC-1α in HepG2 grown in 10 mM galactose as compared with cells grown in 11 mM glucose, whereas a marked significant decrease was seen in cells grown in 25 mM glucose.

The results obtained for HepG2 cells grown in the absence of glucose prove that mitochondrial biogenesis was stimulated and resulted in an increase of mitochondrial mass, probably in order to compensate for the diminished supply of glycolytic ATP—they are in accordance with data obtained by Weber and colleagues using the same cell line [8]. On the contrary, in the hyperglycemic condition, evidence is provided here for the silencing of transcriptional regulatory proteins of mitochondrial biogenesis, without any change in mitochondrial mass.

In agreement with these results, the long-term exposure of HepG2 to a high glucose condition has previously been demonstrated to cause a decrease in mitochondrial content and biogenesis [17].

### 2.2. Oxygen Consumption in Intact Cells

**Figure 2:**
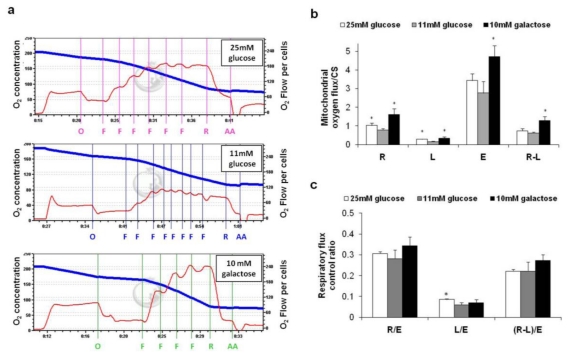
Analysis of oxygen consumption in intact cells. Intact cells were analyzed in complete medium as described in the Methods. (**a**) Representative recordings of oxygen concentration [nmol/mL] (blue line) and oxygen flow [pmol/(s × 10^6^)] (red line) measured by high resolution respirometry and corrected for instrumental background consumption. Abbreviations denote inhibitors and uncoupler added into the chamber (O-Oligomycin, F-FCCP, R-Rotenone, AA-Antimycin A) at the concentrations specified in the Materials and Methods; (**b**) Respiratory data [pmol/(s × 10^6^)], corrected for the rotenone/antimycin A insensitive respiration, were normalized to the mitochondrial mass expressed as CS activity (IU/10^6^ cells). Metabolic states: R-Routine, L-Proton Leak (calculated as the oligomycin-insensitive respiratory rate), R-L (calculated as the difference between the routine and the leak respiration), E-Maximum electron transport system (ETS) capacity (calculated as the maximal respiratory rate in the presence of FCCP). Values are means of three different experiments (bars represent s.d.), * *p* < 0.05 compared with HepG2 cultivated in the presence of 11 mM glucose; (**c**) Normalized respiratory flux control ratios: R/E ratio was calculated as the routine respiration (R) over the maximum ETS capacity (E); L/E was calculated as the leak respiration (L) over the maximum ETS capacity (E); and (R-L)/ E gives the fraction of respiration *vs.* the maximum ETS capacity that is used under routine conditions to produces ATP, referred to as phosphorylating respiration. Values are means of three different experiments (bars represent s.d.), * *p* < 0.05 compared with HepG2 cultivated in the presence of 11 mM glucose.

Oxygen consumption was determined by high resolution respirometry and representative traces are reported in Figure 2a. To evaluate the OXPHOS intrinsic respiratory activity, the respiratory data obtained with the oxygraph were normalized to the mitochondrial mass (CS activity). As reported in Figure 2b, cultivation in aglycemic conditions (black column) led to a significant increase in all the metabolic states of mitochondrial respiration (R, R-L and E), indicating that the entire OXPHOS system was up-regulated upon the cells’ adaptation to glucose-deprivation. Also, this adaptation occurred in excess of the increase of mitochondrial content, and probably involved OXPHOS complex synthesis or activity regulation. In particular, based on the increased value of R-L, which represents the fraction of respiration actually used for ATP production, the data suggest that cells increased their mitochondrial ATP production to survive, in accordance with Marroquin *et al.* [18]. Notably, no changes in the respiratory flux control ratios (R/E, L/E and (R-L)/E) were observed (Figure 2c), indicating that OXPHOS increased the production of ATP without improving the efficiency of the system. These data are in accordance with a recent report that used the HTB 126 breast carcinoma cell line cultivated in aglycemic conditions [6].

On the other hand, cultivation in 25 mM glucose (white column) led to a marked increase in proton leak (L)—measured as oligomycin–insensitive respiration, and in the ratio of proton leak to FCCP-uncoupled respiration (L/E)—as compared with that seen in 11 mM glucose. Strikingly, no significant change in phosphorylating respiration (R-L)/E was observed, documenting that the observed increase in routine respiration (R) occurred to compensate for the increase in uncoupling (L). A more extensive proton leak might be explained by a difference in membrane composition or as the consequence of an increase in the expression of proteins involved in the matrix return of the protons. In this regard, more investigations should be carried out. The uncoupling protein isoform 2 (UCP2) is also worth mentioning as it is an inner mitochondrial membrane protein which contributes to the regulation of the mitochondrial membrane potential and has been found to be overexpressed in various cancer cell lines and in primary human colon cancer [19]. Thus, it could therefore account for an increase in proton leak in such cells.

Finally, the protocol used also allowed us to evaluate the rate of non mitochondrial oxygen consumption as the residual respiration in the presence of rotenone and antimycin A (Table 1). It is interesting that this rate was very high in the hyperglycemic condition, in accordance with the finding by Herst and Berridge that highly glycolytic tumor cells use trans-plasma membrane electron transport (tPMET) to alleviate intracellular reductive stress, and in line with their hypothesis that cell surface oxygen consumption via tPMET may support glycolytic energy metabolism by re-oxidizing cytosolic NADH to facilitate continued glycolysis [20]. Conversely, in aglycemia, the non mitochondrial oxygen consumption was lower and accounted for 14.9% of the total respiration, as was expected on the basis of the up-regulation of mitochondria biogenesis and of the OXPHOS system that occurred in this condition.

### 2.3. Expression Levels of Complex II, ATP Synthase and Inhibitor Factor 1

**Figure 3:**
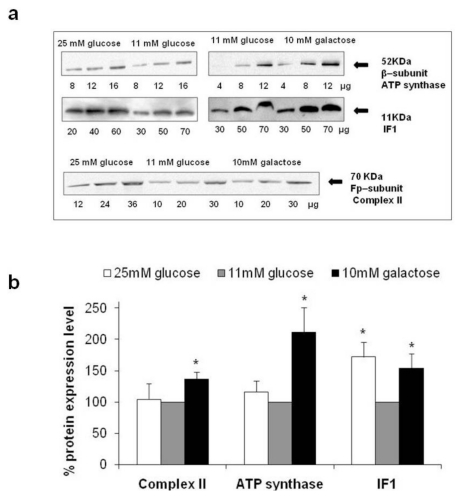
Analysis of Complex II, ATP synthase and Inhibitor Factor 1 (IF1) expression level (**a**) Typical appearance of the immunoreactive bands for the Fp subunit of Complex II, the β subunit of ATP synthase, and IF1 obtained by Western blotting mitochondria from HepG2 cultivated in different glucose concentration; (**b**) Expression levels (%) of Complex II, ATP synthase, and IF1 in mitochondria from HepG2 cultivated in high and no glucose conditions compared to the expression levels detected in cells grown in 11 mM glucose, considered as 100%. For quantification purposes and as loading control, a linear relationship was verified between the band intensities (densitometric analysis) and the protein quantities loaded into the gel to ensure non-saturating conditions and high reproducibility. Values were inferred on the basis of the slope of the straight line and are means of three different experiments (bars represent s.d.); * *p* < 0.05.

Expression levels of the Fp subunit of Complex II, the β subunit of ATP synthase, and its natural Inhibitor Factor 1 (IF1) were examined by quantitative Western blot analysis on crude mitochondrial fractions isolated from HepG2 grown in different glucose concentrations (Figure 3a). The levels of these proteins were determined under non–saturating antibody conditions and the results were summarized in the histograms reported in Figure 3b.

HepG2 cultivated in the absence of glucose showed a significant increase in the expression levels of Fp-Complex II (+36.5%), β-ATP synthase (+111.4%), and IF1 (+53.8%), demonstrating that the improvement of the respiratory capacity observed, resulted from the induction of OXPHOS protein expression, in agreement with previous studies [5,6].

Strikingly, under high glucose conditions, only the IF1 expression level increased (+72.2%), while the expression of Fp-Complex II and β-ATP synthase remained unchanged with respect to levels seen in 11 mM glucose. The different response of ATP synthase and IF1 throughout cell adaptation to an increase in glucose availability, suggests that the expression of the two proteins may be regulated by different and independent pathways, even if the proteins are structurally and functionally closely related. IF1 is a well known reversible non competitive inhibitor of ATP synthase that arrests the functioning of the enzyme when the mitochondrial membrane potential drops [21], playing an important role in tissue ischemia pathology by helping to conserve ATP under conditions of oxygen deprivation. Moreover, it was recently reported that IF1 is able to stabilize the oligomeric forms of ATP synthase [22], which can, in turn, determine mitochondrial cristae shapes [23]. For this reason, we further evaluated whether the increase in IF1 expression levels, observed during hyperglycemia, correlated with an improvement in oligomer stability.

### 2.4. Supramolecular Organization of Mitochondrial ATP Synthase in Membranes

**Figure 4:**
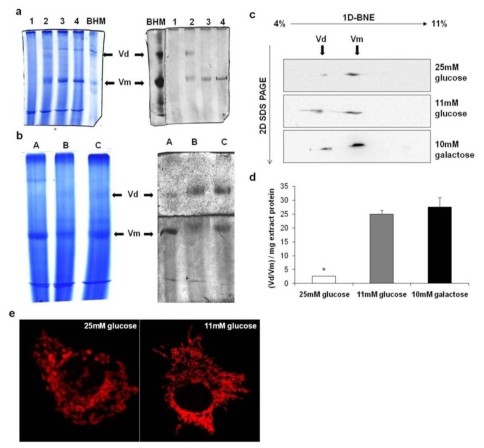
Two-dimensional analysis of monomeric and dimeric forms of ATP synthase and mitochondrial network morphology in HepG2 grown in different glucose concentrations (**a**) Aliquots of mitochondria isolated from HepG2 grown in DMEM containing 25 mM glucose were treated with the indicated concentrations of digitonin (1, 2, 3, 4 μg/μg, dig/prot) and analyzed by BNE Coomassie-stained (left) or stained for in-gel ATPase activity (o/n incubation). BHM: bovine heart mitochondria extract used as a standard, Vd: dimeric form of ATP synthase, Vm: monomeric form of ATP synthase; (**b**) Mitochondrial extracts (optimal concentration of digitonin 2 μg/μg, dig/prot) from HepG2 grown in 25 mM glucose (A), 11 mM glucose (B), and 10 mM galactose (C) were analyzed by BNE Coomassie-stained (left) or stained for in-gel ATPase activity (o/n incubation). The BNE gel was cut before activity staining in order to avoid substrate limitation due to the marked differences in the amounts of dimer and monomer resolved by BNE; (**c**) Monomer and dimer separation was achieved by 1D BNE followed by 2D SDS-PAGE of the single lanes. 2D gels were blotted onto nitrocellulose membrane and then exposed to a monoclonal antibody specific for the β subunit of ATP synthase complex; (**d**) Densitometric analysis of the immunoreactive bands of 2D immunoblotting. The ratio between dimeric and monomeric forms of ATP synthase (Vd/Vm) was normalized per mg of protein in the detergent-extract loaded onto the gel. Values are means of three different experiments (bars represent s.d.); * *p* < 0.05 compared with HepG2 cultivated in the presence of 11 mM glucose; (**e**) Mitochondrial network morphology of HepG2 cultivated in the presence of 25 mM glucose or 11 mM glucose. Confocal images are from one experiment representative of five.

To investigate whether the supramolecular organization of ATP synthase was affected by the bioenergetic adaptation of mitochondria and whether the increase in IF1 expression level was correlated with ATP synthase oligomer stability, we used BNE to analyze mitochondria isolated from HepG2 cultured in different substrates. Detergent extraction of mitochondria was performed with digitonin under controlled conditions, due to its ability to maintain the weak interactions stabilizing dimeric/oligomeric interfaces [24]. Considering that the efficacy of the extraction and the amount of native monomeric and oligomeric ATP synthase resolved by BNE depends on the concentration of detergent used for extraction, we carefully titrated the digitonin in order to identify the optimal concentration of the digitonin to protein ratio for each condition investigated. A representative example of digitonin titration on mitochondria isolated from HepG2 grown in 25 mM glucose is reported in Figure 4a. The dimeric form (Vd) only appeared in gels stained for ATPase activity using 2 μg of digitonin per μg of mitochondrial protein. This digitonin concentration was also verified as being that which allowed the better extraction of the dimeric form under the other conditions and was routinely used. Unfortunately, higher oligomers were not resolved by BNE, irrespective of the digitonin concentration used. We therefore considered the dimer/monomer ratio (Vd/Vm) to be an index of the stability of ATP synthase oligomers.

Figure 4 (panel b and c) shows the results of two-dimensional immunoblotting quantitative analysis of ATP synthase monomers and dimers, resolved by BNE, of digitonized mitochondria from cells grown under different glucose conditions. The first-dimension native gel was stained with Coomassie blue (panel b, left); bands assignment was performed by in-gel development of ATPase activity (panel b, right). BNE lanes were then subjected to a second-dimension SDS-PAGE, followed by immunoblotting of the β subunit (Figure 4c).

The densitometric analysis, reported in the histogram in Figure 4d, showed that the supramolecular organization of ATP synthase in HepG2 cultivated under high glucose condition was markedly worse compared to the cells cultivated in aglycemia, as indicated by lower dimer stability. The dimer/monomer ratio (Vd/Vm), normalized per mg of extracted protein, was 2.7 ± 0.07 for HepG2 grown in 25 mM glucose and 28 ± 3.5 for HepG2 grown in 10 mM galactose.

These results contrast with those obtained by Campanella *et al.* [22], who used a molecular approach, and allow us to exclude the possibility that IF1 plays a role in the stabilization of ATP synthase dimers in our model, as the degree of IF1 over-expression was greater in HepG2 cultured in 25 mM glucose than in aglycemic conditions.

On the other hand, we can conclude that the energy substrate modulates the supramolecular organization of ATP synthase, as it does for the mitochondrial structure. Indeed, in accordance with the paper that first documented an increase in the mitochondrial network in cancer cells cultured in aglycemic conditions [5], Plecita-Hlavata *et al.* have clearly demonstrated using 4Pi confocal microscopy that the mitochondrial network morphology in HepG2 is influenced by the energetic status induced by culture conditions, with mitochondria undergoing rapid fragmentation upon their exposure to high glucose [7]. This may be a result of prolonged reactive oxygen species production [25]. To verify the influence that glucose availability has upon mitochondrial morphology under our experimental conditions and relate it to the effect elicited upon the supramolecular organization of ATP synthase, we examined cells by fluorescence confocal microscopy using the mitochondrial probe Mitotracker Red. In HepG2 cells grown in glucose medium mitochondria appears as a reticulum, mostly clustered with bulkier and more spherical tubules, in contrast to the reticulum in galactose-grown cells that appeared to be more interconnected and ramified (Figure 4e). Thus, it is not unrealistic to link the alteration of the mitochondrial network with the marked alteration of the supramolecular organization of ATP synthase (very low Vd/Vm), shown here under the high glucose condition. We can also hypothesize that the high Vd/Vm ratio observed in aglycemia may be related to a better morphology of the mitochondrial network. Indeed, several lines of evidence suggest a prominent role of ATP synthase in the maintenance of mitochondrial morphology. The cone-shape of the assembled ATP synthase oligomers was proposed to explain the curvature of the inner membrane cristae, and the deletion of subunits, mediating oligomeric interactions, was found to provoke significant alterations to mitochondrial ultrastructure and morphology, with mitochondrial filaments appearing partially fragmented [26].

It should be emphasized that few studies exist in the literature that concern ATP synthase supramolecular organization within the mitochondrial membrane of tumor cells. Nevertheless, they provide important evidence indicating that this organization may be influenced by oncogenic transformation. Based on 2D electrophoresis analysis, a strong reduction in ATP synthase oligomers was reported in transformed fibroblasts compared to normal cells [27]. Furthermore, we recently documented that the Vd/Vm ratio observed in an undifferentiated HCC cell line is lower than that seen in HepG2, which are partially differentiated [13], suggesting a link between the acquisition of the low differentiation-phenotype of HCC and the destabilization of ATP synthase oligomers.

In this context, the results reported in the present paper support the idea that factors destabilizing ATP synthase supramolecular organization may participate in the metabolic adaptation of tumor cells to hyperglycemia. Further future investigations are required to verify whether or not modifications of both lipids (e.g., cardiolipin) and proteins (e.g., cyclophilin D or ATP synthase subunits), possibly involved in the stabilization of the oligomers, may occur as a consequence of metabolic adaptation as well as high-glucose-dependent oxidative stress to mitochondria [17].

### 2.5. Proteomic and Functional Analysis of IF1 Association with ATP Synthase

An oncogenic role of the deregulated expression of IF1 in cancer was recently documented [16], and is also suggested by the possibility that the hypoxia-inducible factor-1α (HIF-1α), a key transcriptional factor that controls crucial features of cancer biology, including angiogenesis, glucose metabolism, cell proliferation and invasion [28], may induce an increase in IF1 protein expression in cancer, as it does in a model of acute hypoxia *in vitro* [29]. Moreover, IF1 may participate in the regulation of tumor cell energetic metabolism by controlling ATP production by mitochondrial ATP synthase and mediating the shift of cancer cells to a state of enhanced aerobic glycolysis [16]. We therefore investigated whether HepG2 adaptation to different glucose availability affected the association of IF1 with ATP synthase.

**Figure 5:**
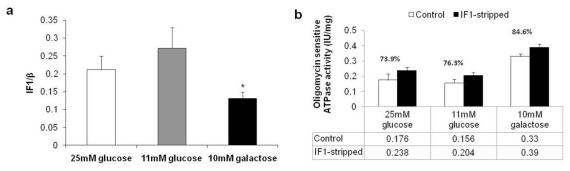
Analysis of the association of the inhibitor protein IF1 to ATP synthase. (**a**) ATP synthase-enriched mitochondrial digitonin extracts were subjected to immunoprecipitation with anti-complex V antibodies and analyzed for IF1 and the β subunit of the complex by immunoblotting. A graphical representation of the IF1/β ratio is shown as obtained by densitometric analysis of the immunoreactive bands. Values are means of three different experiments (bars represent s.d.); * *p* < 0.05 compared with HepG2 cultivated in the presence of 11 mM glucose; (**b**) Oligomycin sensitive ATPase activity (Vmax) was measured on mitochondria permeabilized by osmotic shock after incubation for 35 min at 37 °C in alkaline-high salt conditions (IF1-stripped) or in assay buffer (Control). Values are means of four different experiments (bars represent s.d.); * *p* < 0.05 compared with HepG2 cultivated in the presence of 11 mM glucose.

To establish the state of the association of IF1 with ATP synthase, ATP synthase-enriched digitonin extracts from mitochondrial fractions were subjected to a very sensitive immunocapture procedure [30] with the anti-complex V antibody able to precipitate the complex and associated proteins. Quantification was performed by SDS-PAGE and immunodetection with antibodies recognizing IF1 and the β subunit of ATP synthase as a measure of the complex. Figure 5a shows that no change in the IF1/β ratio was observed in the hyperglycemic condition, indicating that the increase observed in the expression level of IF1 was not correlated with a higher association of the ATP synthase complex. On the contrary, the IF1/β ratio was significantly reduced in the aglycemic condition, indicating a lower degree of enzyme regulation by the inhibitor, despite the increase observed in the expression level of IF1, and in accordance with the finding that such an increase was lower compared with the increase in β-ATP synthase.

Moreover, to validate the immunoprecipitation data, the maximal hydrolytic activity of ATP synthase was measured under activated conditions (*i.e.*, after inducing IF1 release from the complex with a high saline concentration and alkaline pH) and compared with the control condition (*i.e.*, steady-state of IF1 binding). Considering that the alkaline/high salt treatment removes more than 95% of bound IF1 [31], the ATPase activity observed in these conditions was taken as the activity of IF1-free enzyme and considered as 100%. On this basis, we calculated the ratio of enzyme activity between that seen in conditions of steady-state IF1 binding and that of the IF1-free enzyme, expressed as a percentage and used to calculate the amount of IF1-inhibited enzyme. In accordance with the immunoprecipitation data (Figure 5b), we found the percentage of IF1-inhibited enzyme in HepG2 grown in 11 mM of glucose to be 23.7%, which was not significantly different to that seen in the hyperglycemic condition (26.1%). On the contrary, in the aglycemic condition, the amount of IF1-inhibited enzyme was significantly lower at 15.4%. These results confirm that an increase in the IF1 expression level does not correlate with a greater degree of IF1 association to the ATP synthase complex, either in the aglycemic condition (in which IF1 association is lower), or in highly glycolytic cells. Here the steady-state binding of IF1 to ATP synthase is the same as that seen in conditions with lower glucose availability, in line with similar mitochondrial energy production levels in the two conditions. The effect of hyperglycemic or aglycemic conditions on conditioning factors (*i.e.*, pH, membrane potential, calcium) affecting the binding of IF1 to ATP synthase may be the object of further investigations.

## 3. Experimental Section

### 3.1. Cell Culture Conditions

HepG2 human hepatocarcinoma cells were cultured in DMEM (Euroclone) medium containing different glucose concentrations (25 mM and 11 mM); as well as in a galactose medium consisting of DMEM deprived of glucose (Invitrogen), supplemented with 10 mM galactose. All mediums also contained 1 mM pyruvate, 4 mM glutamine, 10% FSC, 100 U/mL penicillin, and 100 μg/mL streptomycin. Whereas glutamine is used to produce ATP through OXPHOS, the galactose goes mainly to the pentose phosphate pathway. Frozen HepG2 cells, previously grown in 11 mM glucose medium, were thawed and grown for 8 days in high glucose or galactose medium and used for experiments when approaching 90% confluence. Cells were kept in 5% CO_2_ at 37 °C at air saturation. Cell proliferation was evaluated daily for 8 days. Doubling time (DT) was calculated from the exponential growth period as follows: DT = (T2 − T1)/[ln(cell number at T2/cell number at T1)/ln(2)], where (T2 − T1) is the duration of the exponential phase.

### 3.2. Determination of Mitochondrial Mass

Activity of the mitochondrial matrix enzyme citrate synthase (CS) was assessed in cell homogenates to provide an estimate of mitochondrial mass. CS activity was recorded spectrophotometrically at 412 nm using a UV/Vis Spectrophotometer Lambda14 (Perkin-Elmer), as previously described [13]. Briefly, a background rate was obtained by adding cells, sonicated in 1 M Tris/HCl (pH 8.1), to 1 mM 5,5'-dithiobis-2-nitrobenzoate (DTNB) and 10 mM acetyl-coenzyme A. This initial rate was subtracted from the rate obtained upon the addition of the substrate (10 mM oxaloacetate). Enzyme activities were expressed as IU (μmoles/min) per mg of protein. The yield of the crude mitochondria fractions (see below) from cells suspended at 2 × 10^7^ cells/mL was evaluated after culturing with different substrates and expressed as mg of mitochondrial protein per 10^6^ cells.

### 3.3. Polarographic Measurement of Respiration

Mitochondrial oxygen consumption, measured in intact cells under conditions of physiological substrate supply, was performed at 37 °C using a high-resolution respirometer Oxygraph 2 k (Oroboros instruments, Innsbruck, Austria). Routine respiration (R) was measured in 3 × 10^6^ cells in 3 mL chambers containing culture medium, while the leak respiration (L) was obtained in the presence of oligomycin (2 μg/mL), which inhibits ATP synthase; consequently, the electron flow reflects the energy requirement to compensate the futile circle of proton pumping. The maximal uncoupler–stimulated respiratory activity (E), measured in the presence of a concentration of the uncoupler FCCP (carbonyl cyanide *p*-trifuoromethoxy-phenylhydrazone) empirically determined as optimal (2.5 μM), provides a measure of the capacity of the electron transport system (ETS). 1 μM rotenone (which inhibits complex I) and 2.5 μM antimycin A (which inhibits complex III) were used to determine the non mitochondrial oxygen consumption. This rate was subtracted from cell total oxygen consumption to assess the mitochondrial respiration. Using ETS capacity as a common basis for the normalization of the metabolic states, the R/E ratio reflects the level of mitochondrial activity relative to ETS capacity. Correspondingly, the L/E ratio reflects the level of leak respiration relative to the ETS capacity and provides an estimate of intrinsic uncoupling. Finally, the fraction of respiration actually used for ATP production (phosphorylating respiration) is estimated as the difference between R/E and L/E, and indicated as (R-L)/E. Data were digitally recorded using DatLab4 software; oxygen flux was calculated as the negative time derivative of the oxygen concentration, cO_2_(t). A standard correction was performed for instrumental background oxygen flux arising from oxygen consumption of the oxygen sensor and minimal back-diffusion into the chamber.

### 3.4. Preparation of Crude Mitochondria Fractions

Collected cells were suspended at a concentration of 2 × 10^7^ cells/mL in mitochondria isolation buffer containing 250 mM sucrose, 1 mg/mL BSA, 2 mM EDTA (pH 7.4), and protease inhibitor cocktail (1:100), and sonicated at ice-cold temperature (5 s bursts were repeated 3 times with 30 s intervals). The resultant homogenate was subjected to differential centrifugation: 800 g for 20 min at 4 °C. The supernatant was centrifuged at 16,000 g for 20 min at 4 °C to obtain the crude mitochondrial fraction as a pellet. The pellet was resuspended in buffer solution containing 250 mM sucrose, 10 mM Tris-HCl, and 0.1 mM EGTA (pH 7.4) and used immediately for enzymatic analysis or stored at −80 °C.

### 3.5. Western Blot Analysis of Cell and Mitochondrial Lysates

The preparation of samples from cell lysates and crude mitochondrial fractions and electrophoresis were performed as described previously [13] and subjected to Western blot analysis using the following antibodies: mouse monoclonal anti-IF1 (Mitosciences), rabbit polyclonal anti-β subunit of ATP synthase (Mitosciences), mouse monoclonal anti-Fp subunit of Complex II (Mitosciences) and rabbit polyclonal anti-PGC1α (Abcam). Densitometric analysis was performed with Quantity One 4.2.1 software (Bio-Rad Hercules, California). A linear relationship between increasing band intensities and the quantities of proteins loaded into the gel was confirmed in each case in order to prove non–saturating conditions. Quantitative data were inferred by densitometric analysis of immunoreactive bands on the basis of the slope of the straight line and reported as % of HepG2 grown in 11 mM glucose expression level, taken as 100%.

### 3.6. 2D-Immunoblotting Analysis with Blue Native Electrophoresis as a First-Dimension

Pellets of heavy mitochondria from bovine heart (BHM), isolated as described in Tomasetig *et al.* [32], and HepG2 crude mitochondria fractions were suspended at 6.25 mg/mL and 15 mg/mL, respectively, in extraction buffer (5 mM aminocaproic acid, 50 mM NaCl, and 30 mM Tris pH 7.4), solubilized with different concentrations of digitonin (2.4 μg/μg dig/prot for BHM and 1, 2, 3, 4 μg/μg dig/prot for cell mitochondria), and immediately centrifuged at 100,000 *g* for 25 min at 4 °C. Titration curves were created to establish the optimal detergent amount to extract intact ATP synthase complex in its monomeric and oligomeric forms. The protein digitonin extracts, quantified using the Bradford method, were supplemented with Coomassie Blue G-250 (Serva) and rapidly applied to 4–11% polyacrylamide gradient blue native gel (BNE). After electrophoresis, gels were stained with Coomassie Blue or used for in-gel activity staining. Coomassie Blue-stained gels were quantified by densitometry using ImageQuant software, version 2003.03 (Amersham Biosciences). Gel strips from BNE were further resolved by two-dimensional SDS-PAGE (17%), followed by Western blotting using anti-β subunit antibody (Mitosciences).

### 3.7. Confocal Microscopy

5 × 10^4^ cells were plated in complete medium on glass coverslips and grown overnight. Cells were stained with the mitochondrial marker Mitotracker Red CMXRos (100 nM) (Molecular Probes, Eugene, OR, USA) in complete medium. After staining, cells were fixed in medium containing 3.7% paraformaldehyde and analysed using a laser scanning confocal microscope equipped with a 488–534 λ Ar laser and a 633 λ He-Ne laser (Leica TCSNT, Leica Mycrosystem, Wetzler, Germany).

### 3.8. ATP Synthase Immunoprecipitation and IF1 Immunodetection

Mitochondrial extracts, obtained as described above, were used to immunoprecipitate ATP synthase and immunodetect IF1 bound to the enzyme. Aliquots were incubated overnight under wheel rotation at 4 °C in the presence of anti-complex V monoclonal Ab covalently linked to protein G-Agarose beads (MS501 immunocapture kit from Mitosciences) in a ratio of 20 μL/mg of protein, as recommended by the immunocapture kit supplier. After gentle centrifugation (500 g for 5 min), the beads were washed twice for 5 min in a solution of 0.05% (w/v) *n*-Dodecyl-β-d-maltoside (DDM) in PBS. The elution was performed in 2% (w/v) SDS for 15 min, and the collected fractions were subjected to SDS-PAGE, followed by Western blotting using anti-β subunit of ATP synthase and anti-IF1 antibodies.

### 3.9. Mitochondrial ATP Synthase Activity

Maximal ATPase activity (Vmax) of mitochondrial ATP synthase was measured in an ATP-regenerating system at 37 °C. Mitochondrial membranes obtained by osmotic shock (incubation dilution 1:5 for 3 min) were incubated in a medium containing 30 mM sucrose, 50 mM Tris/HCl (pH 7.4), 4 mM MgCl_2_, 50 mM KCl, 2 mM EGTA 2 mM phosphoenolpyruvate, 0.3 mM NADH, 2 μg/mL rotenone, 4 IU of pyruvate kinase, and 3 IU of lactate dehydrogenase. The reaction was started by the addition of 2.5 mM ATP and the rate of NADH oxidation, equimolar to ATP hydrolysis, was monitored as the decrease in absorbance at 340 nm. To induce the release of the inhibitor protein IF1 from ATP synthase, the mitochondrial membranes obtained by osmotic shock were incubated for 35 min at 37 °C under “activating conditions” consisting of 125 mM KCl, 2 mM EDTA, and 30 mM Tris-SO_4_ (pH 8.5). The ATPase activity sustained by ATP synthase was measured on the basis of its sensitivity to 10 μM oligomycin. Results are reported as IU/mg; one unit was defined as that required for the oxidation of 1 μmol NADH/min/mg.

### 3.10. Statistical Analyses

The results presented correspond to mean values ± standard deviations (s.d.); Student’s *t* tests were used to test for statistically significant differences; *p* < 0.05 was considered to be statistically significant.

## 4. Conclusions

This work investigated the effects induced on mitochondria by changes in glucose availability in the HepG2 hepatocarcinoma cell line, mainly focusing on ATP synthase and Inhibitory Factor 1. Aglycemic culture conditions stimulated mitochondrial activity in HepG2 cells as a result of increased mitochondrial mass and OXPHOS protein expression levels, demonstrating the OXPHOS system to play an important role in energy production and cell viability under glucose–limiting conditions. Notably, the regulation of ATP synthase by IF1 was reduced.

We demonstrated, for the first time, a hyperglycemia-induced alteration in the supramolecular organization of ATP synthase. We propose this as the probable cause of the extensively reported glucose-induced modulations to mitochondrial morphology [5–7,33]. To our knowledge, the impact of metabolic adaptation on the supramolecular organization of ATP synthase has not been previously described. Furthermore, we demonstrated a hyperglycemia-induced increase in IF1 expression levels, whilst ATP synthase levels remained unaltered. IF1 overexpression in tumor cells has previously been documented and linked to the more efficient regulation of ATP synthase [13,16,34]. Surprisingly, in our model, an increase in mitochondrial IF1 did not lead to any impairment in the phosphorylation rate and was not associated with an increase in the steady-state binding of IF1 to ATP synthase. In conclusion, the deregulated expression of IF1 in tumor cells induced by hyperglycemia may exert an effect not related to the well known role of IF1 on ATP synthase regulation, or may represent the cells’ effort to control ATP synthase activity during stressful conditions (e.g., hypoxia).

The overall clinical significance of our findings is unclear and requires additional work to improve the understanding of the role of hyperglycemia-induced deregulation of IF1 expression and alteration in the supramolecular organization of ATP synthase, as well as the role of up-regulated OXPHOS system in energy production and cell viability under glucose–limiting conditions. Nevertheless, our findings provide some new information that may be useful for comprehension of the oncogenic role of deregulated IF1 expression (mimicked under hyperglycemic conditions), together with the putative oncosuppressor role of mitochondrial biogenesis and ATP synthase supramolecular organization (mimicked under aglycemic conditions) in relation to the understanding of cancer metabolic adaptation and the development of future targeted therapeutic strategies.

## Figures and Tables

**Table 1:** Non mitochondrial *vs.* mitochondrial oxygen consumption. The rate of oxygen consumption was detected in intact cells under condition of physiological substrate supply referred to as routine respiration (R). Measurements were performed in the presence of 1 μM rotenone and 2.5 μM antimycin A to determine the non mitochondrial oxygen consumption and this rate was subtracted from that of total oxygen consumption to assess the mitochondrial respiration.

	Total O_2_ Consumption	Mitochondrial O_2_ Consumption	Non Mitochondrial O_2_ Consumption
**25 mM Glucose**	73.3 ± 11.7 (100%) *	41.1 ± 5.5 (56.2%) *	32.1 ± 6.5 (43.8%) *
**10 mM Galactose**	107.7 ± 24.6 (100%)	91.7 ± 17.2 (85.1%)	16 ± 7.8 (14.9%)

Values are expressed as [pmol/(s × 10^6^)] and are means ± s.d. of three different experiments;

* *p* < 0.05 compared with HepG2 cultivated in the presence of 10 mM galactose.
